# High-throughput metabolomics and ingenuity pathway approach reveals the pharmacological effect and targets of Ginsenoside Rg1 in Alzheimer’s disease mice

**DOI:** 10.1038/s41598-019-43537-4

**Published:** 2019-05-07

**Authors:** Ge Li, Ning Zhang, Fang Geng, Guoliang Liu, Bin Liu, Xia Lei, Guang Li, Xi Chen

**Affiliations:** 1 Yunnan Branch, Institute of Medicinal Plant Development, Chinese Academy of Medical Sciences & Peking Union Medical College, Xuanwei Avenue 138, Jinghong City, 666100 Yunnan Province China; 2 College of Jiamusi, Heilongjiang University of Chinese Medicine, Jiamusi, Guanghua Street 39, Qianjin District, Jiamusi City, 154007 Heilongjiang Province China; 3 College of Chemistry & Chemical Engineering, Harbin Normal University, Shida Road No. 1, Limin Economic Development Zone, Harbin, 150025 Heilongjiang Province China; 4 Institute of Medicinal Plant Development, Chinese Academy of Medical Sciences & Peking Union Medical College, Beijing, 100193 China

**Keywords:** Metabolomics, Mass spectrometry

## Abstract

Ginsenoside Rg1, a natural triterpenoid saponins compound isolated from the Panax species, has been found to possess neuroprotective properties in neurodegenerative diseases such as Alzheimer’s disease (AD). However, its pharmacological mechanism on AD has not been studied. In this study, an ultra-performance liquid chromatography combined with quadrupole time of-flight mass spectrometry (UPLC-Q/TOF-MS) based non-targeted metabolomics strategy was performed to explore the mechanism of Ginsenoside Rg1 protecting against AD mice by characterizing metabolic biomarkers and regulation pathways changes. A total of nineteen potential metabolites in serum were discovered and identified to manifest the difference between wild-type mice and triple transgenic mice in control and model group, respectively. Fourteen potential metabolites involved in ten metabolic pathways such as linoleic acid metabolism, arachidonic acid metabolism, tryptophan metabolism and sphingolipid metabolism were affected by Rg1. From the ingenuity pathway analysis (IPA) platform, the relationship between gene, protein, metabolites alteration and protective activity of ginsenoside Rg1 in AD mice are deeply resolved, which refers to increased level of albumin, amino acid metabolism and molecular transport. In addition, quantitative analysis of key enzymes in the disturbed pathways by proteomics parallel reaction was employed to verify changed metabolic pathway under Ginsenoside Rg1. The UPLC-Q/TOF-MS based serum metabolomics method brings about new insights into the pharmacodynamic studies of Ginsenoside Rg1 on AD mice.

## Introduction

Alzheimer’s disease (AD) is an irreversible degenerative nervous system disease, which is the major cause of dementia in elderly individuals around the world^1,2^. It is characterized by extracellular senile plaques in brain consisted of β-amyloid (Aβ) peptides, intracellular neurofibrillary tangles, neuronal loss, and synapse injury in some vulnerable regions such as the hippocampus^3–5^. Approximately 24 million population worldwide with dementia and over 50%-70% of dementia cases attribute to AD. With the global population rapidly increasing, 115 million people will have AD by the year 2050 according to the World Alzheimer Report 2016. An astonishing health-care burden we face is the expense of dementia have increased from 604 billion in 2010 to 818 billion in 2015 and possibly up to 1 trillion within three years^6^. Nowadays, the main available measure to resist AD is drug therapy, which aim to relieve pathological symptoms of sufferers, preventing or slowing down AD progressing using symptomatic therapy, biological therapy and etiological therapy. Only five drugs were ratified by Food and Drug Administration(FDA) to cure AD in clinical treatment, including Donepezil, Rivastigmine Hydrogen Tartrate, Galanthamine, Huperzine-A, Memantine. Before symptoms of the disease emerge, individual had be in pathophysiological state of AD for many years^7,8^. Although significant therapeutic interventions and pharmaceutical researches of resisting AD have been accomplished in the past decade, most AD patients remain dismal in therapeutic process. Currently, there is an urgent and recognized need for exploring and establishing effective treatment strategies against AD.

From more than two thousand years, Traditional Chinese medicine (TCM) had applied in the Chinese public health system, and a larger amount of active pharmacological ingredient from herbal medicines had been separated and validated to have potential in improving memory^9,10^. Compared with western medicine, they are considered as promising anti-AD drugs to relieve dementia and neurodegenerative syndromes with high efficacy and fewer toxic or side effects for clinical therapy, such as Ginkgo biloba, Turmeric, Cornus and Rhodiola rosea^11–14^. Among the effective herbs, ginseng improves the ability to combat stress, trauma, anxiety and fatigue, cancer and neurodegenerative diseases prevention in middle-aged and elderly populations^15^. Ginsenoside Rg1, an active small-molecule compounds purified from ginseng, could penetrate the blood-brain barrier and possess a positive effect on the brain such as improving cognitive impairment and memory^16^. Several studies revealed that Rg1 decrease the levels of cerebral Aβ, alleviate oxidative stress, enhance the scavenging of free radicals, inhibit cellular apoptosis induced by Aβ accumulation, keep neuron activity and plasticity within a normal state in hippocampus in animal model of AD^17^. But, the underlying mechanisms of Rg1 on inhibiting pathological changes of AD in metabolic level was little known.

Dated from the Greek word “to change”, metabolism comes down to all chemical reactions in cells, which is characterized by synchronized catabolic and anabolic processes in the healthy state. With the development of systems biology, metabolomics as an advanced profiling technique *in vivo* might offer a novel platform to interpretate the situation of multiple endogenous metabolites changes in response to endogenous and exogenous stressor^18–20^. It places emphasis on systematically and holistically probing into the metabolome and metabolic pathway variation by the identification of metabolites and measurement of metabolite concentration. Nowadays, metabolomics has been successfully applied to detect metabolic changes in AD and to characterize biochemical pathways. It contributes to identify biomarkers in early stage of AD, to seek emerging therapeutic targets, and to detect therapeutic response and disease progression^21–24^. Quantitative metabolite data sets analysis can be employed to bring hypotheses associated with mechanisms of disease pathogenesis or potential targets of drug as well as gene function into being. In consideration of the considerable similarity between mouse and human metabolism, the application of metabolomics offers appropriate translation of animal research into human studies for speeding up drug design, especially natural product^25,26^. The aim of this study takes the triple transgenic AD mice as objects, to preliminarily discuss the capability of the metabolomics approach and explore the molecule mechanism of Ginsenoside Rg1 in treating AD, which attempt to bring out preliminary basic research for drug development of natural resources in AD clinical treatment.

## Material and Methods

### Materials

Acetonitrile and methanol in HPLC grade were obtained from Merck (Darmstadt, Germany). Formic acid in HPLC grade was supplied by Fisher Scientific Corporation (Trinidad). Distilled water used to prepare mobile phase and the aqueous solutions was offered by Watsons Food & Beverage Co., Ltd. (Guangzhou, China). Pentobarbital sodium and physiologic saline solution were obtained from Sigma Aldrich (Saint Quentin Fallavier, France). Leucine enkephalin were obtained from Sabinsa Corporation (Piscataway, USA). Ginsenoside Rg1 with a molecular weight of 801.01 was purchased from Hongjiu Biotech. Co., Ltd. (Jilin, China) in the form of white powder-like crystals, general formula C_42_ H_72_O_14_, and the purity more than 99% was measured by HPLC. The assay kit of phospholipase A2, cytochrome p450, the enzyme prostaglandin-F synthase, PR-SET7 and indoleamine 2,3-dioxygenase 2 were obtained from Sinopharm Chemical Reagent Co., Ltd. (Shanghai, China). Other reagents and chemicals were of analytical grade during the stage of experiment.

### Animals and treatment

All animal experiments were performed according to relevant national legislation and local guidelines. The male wild type (strain:B6129SF2/J) mice and 3xTg-AD mice which harbor PS1_M146V_, APP_Swe_, and Tau_P301L_ transgenes in seven-week-old were purchased from the animal center of the National Institute for the Control of Pharmaceutical and Biological Products (Beijing, China). The experimental procedures were approved by the Animal Care and Ethics Committee at Chinese Academy of Medical Sciences & Peking Union Medical College and all experiments were performed in accordance to the declaration of Helsinki. After behavioral and pathological test, 3xTg-AD mice is verified to possess the AD mouse model traits. They were housed in animal room for acclimatization one week with a 12 h light/dark cycle at constant temperature of 24 ± 2 °C and humidity of 50 ± 5% and provided standard mouse chow and tap water every day. Then, mice were divided into control group, model group and Rg1-treated group with eight animals for each group. The 3xTg-AD mice in treatment group were treated with Rg1 (16 mg/kg body weight one day) by intragastric administration for 12 weeks, meanwhile, non-treated 3xTg-AD mice in model group and wild type mice in control group were raised with same volume of normal saline^27^.

### Sample collection and preparation

After intragastric administration of Ginsenoside Rg1 for 12 weeks, mouse were anesthetized by intraperitoneal injection of 3% sodium pentobarbital (2 mL/kg) at the next day. Blood samples were collected by abdominal aorta from every group. The anesthetized mice thoracic cavity was dissected and the heart was exposed. After the hemostat was clamped to the abdominal aorta, the aorta was inserted from the left ventricle with a syringe of 50 mL of a sharpening needle, and 50 mL of physiological saline was pushed into it within 1 minute. The brain was quickly taken after perfusion, and the hippocampus tissue was immersed in 10% neutral paraformaldehyde for pathology.

When the blood was drawn, it was firstly placed on ice for at least an hour and then centrifuged at 4500 g for 15 min at 4 °C. The separated supernatants were collected to eppendorf tubes and stored at −80 °C until metabolomics analysis. Before the instrument analysis, the serum samples were thawed at room temperature. Then, moved out 200 μL serum samples and added with 10 μl phosphoric acid, vortexed for 30 s and placed at 4 °C for 10 min. The mixed solution was used to preactivated OASIS HLB SPE columns(Waters, USA), which was successively washed with 3 mL methanol and 3 mL water. Serum samples was eluted by 1 mL water and the eluent was threw away. Then, secondary eluent performed by 100% methanol was dried under N_2_ at room temperature. 150 μL acetonitrile was used to re-dissolve the residues, then the mixed solution was centrifuged at 10,000 g for 10 min at 4 °C. Finally, took the supernatant filtered through 0.22 μm filter membrane, all serum samples were entered into instrument for metabolomics analysis by 5 µL injection volume.

### Instrument method of UPLC-Q/TOF-MS

Chromatographic analysis of serum samples was performed in Ultra Performance liquid chromatography (UPLC), which equipped with apump, autosampler, a 0.17 μm ACQUITY BEH C18 chromatography column (100 mm × 2.1 mm i.d.). An aliquot of 5 μl of serum sample solution was injected for separation at 40 °C colume temperature with the flow rate of 0.5 mL/min. For supervising the stability of the instrument to ensure the consistent performance of the system, a quality control (QC) sample containing 10 μL of each serum sample from different groups was prepared. After chromatography parameters optimization, the mobile phase consisted of a linear gradient system including acetonitrile containing 0.1% formic acid (phase A) and water with 0.1% formic acid (phase B). The elution gradient program was set as follows: 0–2 min, isocratic 1% A; 2–5 min, linear gradient from 1% to 45%A; 5–7 min, 45% to 80% A; 7–8 min, 80% to 100% A; 8–10 min, isocratic 100% A, and afterward back to1% A in 4 min. A needle wash cycle is indispensable during the metabolomics analysis for the purpose of sweeping away the remains and the next injections preparation. The obtained elution was promptly delivered to the MS system before split.

Mass spectrometry (MS) detection of metabolites was performed by a quadrupole time-of-flight mass spectrometer (Xevo G2 Q-TOF MS) (Waters Corporation, Milford, MA, USA), which was equipped with an electrospray ionization source operating in both positive ionization mode (ESI^+^) and negative ionization mode (ESI^−^). The optimal conditions of analysis were as follows: the cone voltage is 45 V in positive mode and 35 V in negative mode, the sampling capillary voltage is 3.5 kV in both modes.The source temperature was set at 120 °C, desolvation gas temperature was 320 °C, cone gas flow was 45 L/h, and desolvation gas flow was 550 L/h. Using 0.15 ng/mL of leucine enkephalin as an external reference (Lock-Spray) under a flow rate of 120 μL/min, data were centroided and the mass was revised in positive ion mode ([M + H]^+^ = 556.2771) and for negative ion mode ([M−H]^−^ = 554.2614). During metabolite profiling, centroid data were gained by MassLynx (V4.1) software for each sample from 50 to 1000 Da with a 0.2 s scan time and a 0.1 s interscan delay over a 10 min run time. The analysis parameters are set as following: fragment voltage of 75 V, collision energy of 30 V, cell accelerator voltage of 25 V. Nitrogen was applied for drying and collision gas.

### Data processing and statistical analysis

All the files of complicated original dataset from control, model and Rg1-treated group were firstly imported and uploaded to Progenesis QI v1.0 software (Nonlinear Dynamics, Newcastle, U.K) for noise reduction, height intensities peak detection, removal of isotope masses and alignment of retention time (rt) and mass (m/z). Then, the resultant data matrices were imported into EZinfo 2.0 software for multivariate analysis such as the principal component analysis (PCA) and orthogonal partial least squares discriminant analysis (OPLS-DA). PCA, as an unsupervised method, is used to reduce variable and perform classification. A supervised OPLS-DA analysis has ability to maximize class discrimination that is conducive to seek potential metabolic biomarkers. In light of the contribution to variation and correlation of OPLS analysis, S-Plots and VIP-plot were obtained to highlight the relationship between covariance and pertinence.

Variables met VIP value > 1 and p value less than 0.05 were regarded as the valuable differentially expressed potential biomarkers between wild type mice and AD mice. Metabolite peaks are assigned and affiliated by MS/MS analysis and MassFragment TM application manager (Waters Corp., Milford, USA). The biomakers information of molecular weight, molecular formula and chemical structures was provided via available biochemical databases online such as ChemSpider, Human Metabolome Database, MassBank.jp (http://www.massbank.jp) and LIPIDMAPS. The pathway analysis of identified metabolites were performed in MetaboAnalyst 4.0, and IPA software containing functions, pathways and network models establishment was used for network enrichment analysis after Ginsenoside Rg1 treatment.

### Targeted protein verification analysis

Protein was extracted from the brain tissue for quantitative analysis according to the instructions of sample preparation kit and protein assay kit. After protein resolved into polypeptides by trypsin, they were resuspended in 0.1% (v/v) formic acid for UPLC-MS analysis. The parallel reaction monitoring analysis of peptides was performed combined Thermo EASY-nLC 1200 nanoliquid chromatography (Thermo Fisher Scientific) by C18 column(75 μm × 15 cm) with SCIEX Qtrap 5500 (SCIEX, Framingham, MA, USA). The UPLC mobile phases were consisted of 100% water with 0.2% formic acid (phase A) and 97% acetonitrile with 0.2% formic acid (phase B), which the gradient was employed as follows: 0–20 min, 0–40% B; 20–40 min, 40–55% B; 40–50 min, 55–80% B; 50–60 min, 80%-100% B. The MS cycle contain a full MS1 scan with 60,000 resolution and the predetermined targeted MS2 scan with the resolution 35,000. For further resolving and calculation, the original data were analyzed using Skyline software (v3.6, MacCoss Lab, Seattle, WA, USA) under a certain condition that peptide enzyme was deemed as Trypsin; peptide length was allowed from 6 to 23 amino acid residues; max missed cleavage was set zero; carbamidomethyl on cys and oxidation on Met; 0.04 Da as the ion match tolerance; precursor charges were set as 2 and 3.

## Result

### Histopathological examination

**Figure 1:**
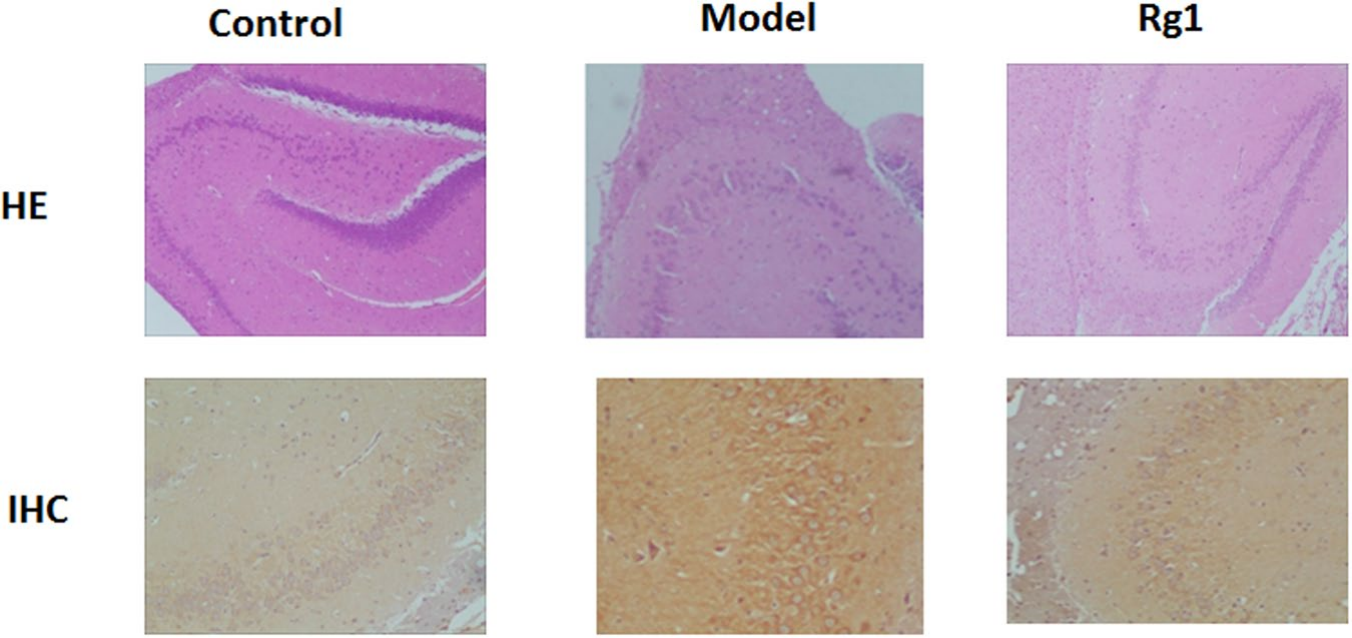
The result of HE staining and immunohistochemistry (IHC) in hippocampal CA3 region of mice brain.

As shown in Fig. 1, in the control group, the hippocampal neurons in the brain tissue were closely arranged, the shape was regular and the number was large, the cell body was full and the nucleolus was clear. In the model group, the nucleus of the neurons showed pyknosis. A large number of neurons were missing and the boundary of the nuclear membrane was blurred. Compared with the model group, the neurons in the hippocampal CA3 area was more and the cell body was fuller after Rg1 intervention which indicates that Rg1 slows down the neuronal cell atrophy caused by modeling. The Aβ1–40 response in the brain tissue of the control group was negative, and the positive reaction substances in the AD model group were mostly concentrated in the neuron cell body, the number of positive plaques was more. Compared with the model group, the number of Aβ1–40 plaques in the Rg1 group was reduced, but still higher than the level of the control group.

### Global analysis of the dynamic metabolic profiling

**Figure 2:**
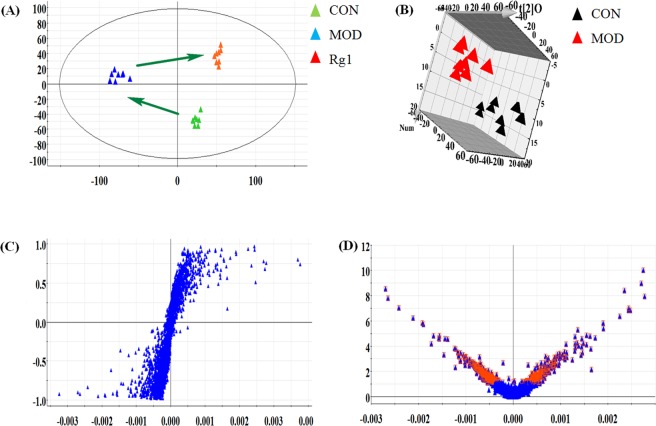
Multivariate data analysis of the serum samples from different groups in positive mode. (**A**) The PCA score plot among control, model and Rg1-treated groups. (**B**) The OPLS-DA score plot between control and model group. S-plot (**C**) and VIP-score plot (**D**) of OPLS-DA between control and model group. Red area represents indicates the ions with VIP >1.

**Figure 3:**
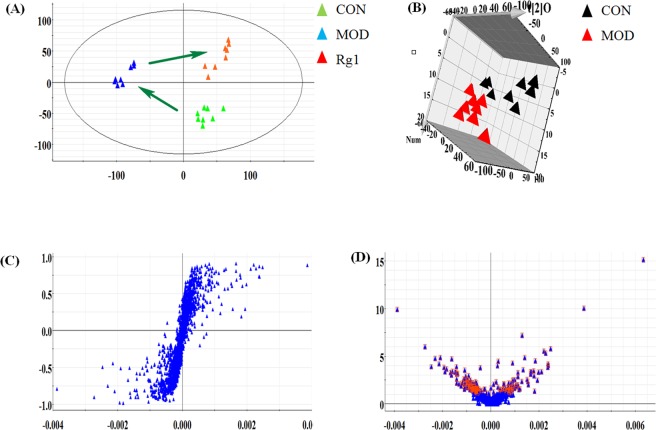
Multivariate data analysis of the serum samples from different groups in negative mode. (**A**) The PCA score plot among control, model and Rg1-treated groups. (**B**) The OPLS-DA score plot between control and model group. S-plot (**C**) and VIP-score plot (**D**) of OPLS-DA between control and model group. Red area represents indicates the ions with VIP >1.

Small molecular mass metabolites in serum sample were separated well in the short time of 10 min. Multivariate statistical analysis was acquired to better understand the differences among these complex data in control, model and Rg1-treated group. As shown in Figs 2A and 3A, the 2D-PCA score plot present a clear trend of trajectories separation in three groups, and the Rg1-treated group trajectory is more close to control group, which reveals that the metabolic discrepancy have occured in control, model and Rg1-treated group, and Ginsenoside Rg1 facilitates model mice from disorder to normal state. For further differentiating between the wild type mice and AD mice to pinpoint Ginsenoside Rg1 activity, the supervised 3D OPLS-DA from Figs 2B and 3B were employed to divide samples into two part. Moreover, the further away ions from the origin have the higher contribution to the chaos in the metabolic trajectory and are likely to be deemed as the potential biomarkers in S-plot (Figs 2C and 3C). From the VIP plot, the significant variables met objective indicators that p values < 0.05 and VIP >1 were used for discrepant metabolites visualization and discovery (Figs 2D and 3D).

### Potential biomarkers identification

**Figure 4:**
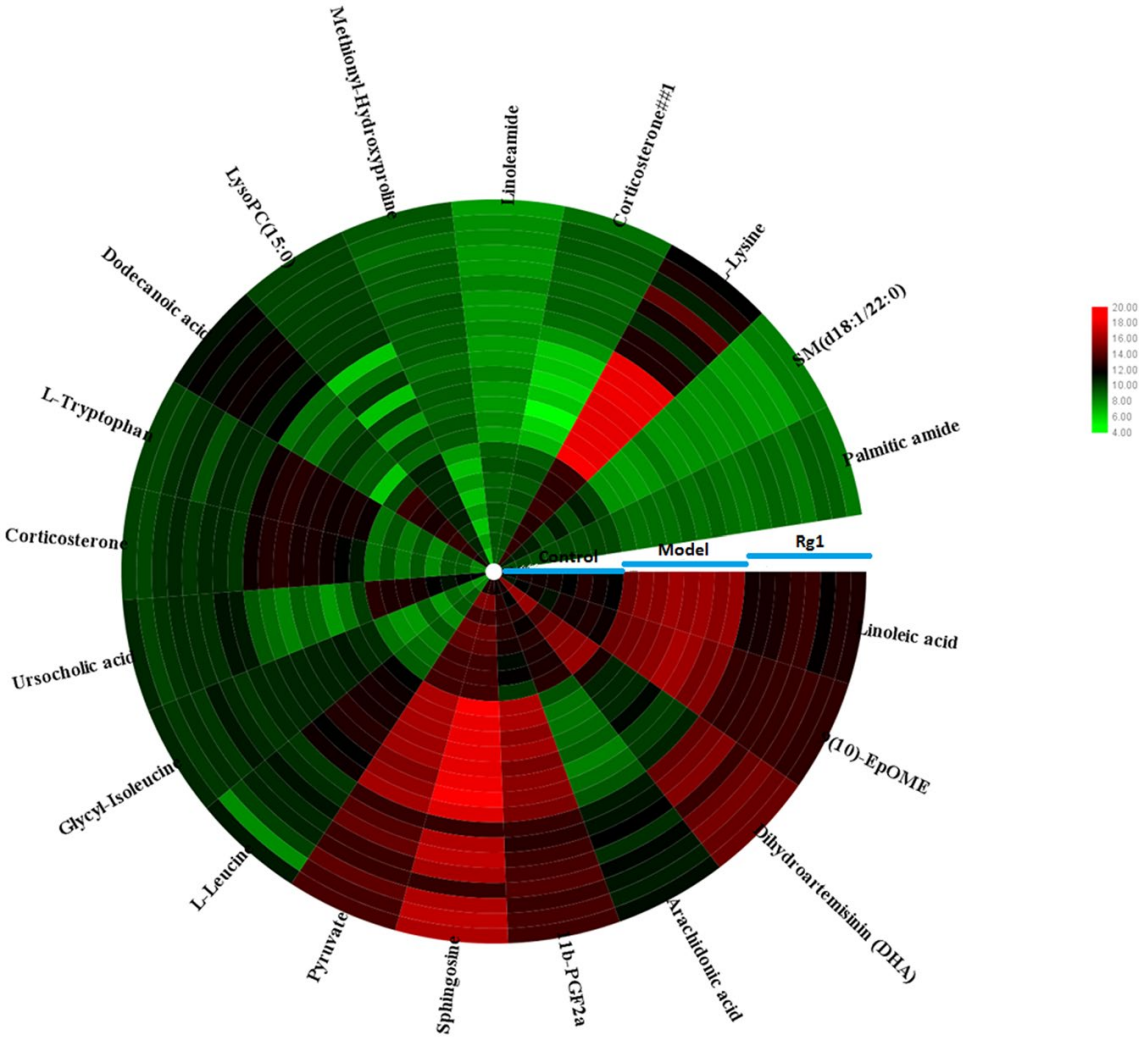
The circular heatmap analyzed showed the concentration evaluation of serum compounds in control, model and Ginsenoside Rg1-treated groups.

**Figure 5:**
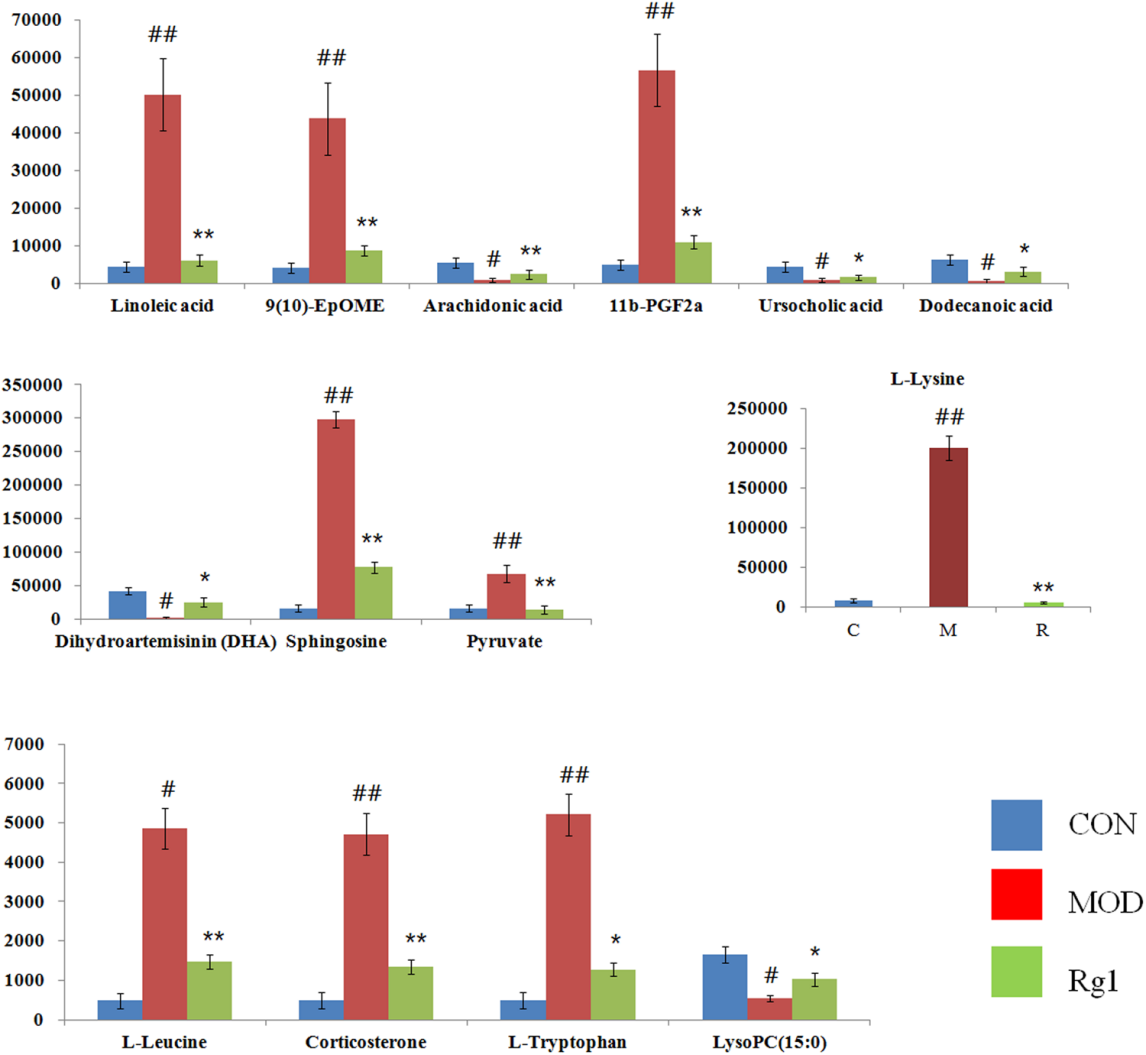
The relative intensity of serum metabolites closely related with Ginsenoside Rg1 treatment of AD. “#” stands for significant difference (p < 0.05) compared by control groups, “##” stands for very significant difference (p < 0.01) compared by control groups. “*” stands for significant difference (p < 0.05) compared by model groups, “**” stands for very significant difference (p < 0.01) compared by model groups.

Firstly, the candidate metabolites were preliminarily identified by the data of accurate mass and MS/MS fragments provided by Q-TOF platform. Then, the information of the potential biomarkers is acquired by consulting online database such as HMDB, METLIN, Chemspider and KEGG. The Rt–m/z pairs of selected differential ions in each groups was matched and distinguished using the tandem MS fragmentation with the reference standards and the database. If a mass difference between the realistic and theoretical mass less than10 ppm, the metabolite name was reported. A total of 19 potential metabolites were characterized related to the AD morbidity between the control and model group (Table S1), including linoleic acid, 9(10)-EpOME, dihydroartemisinin (DHA), arachidonic acid, 11b-PGF2a, sphingosine, pyruvate, L-leucine, glycyl-Isoleucine, ursocholic acid, corticosterone, L-tryptophan, dodecanoic acid, LysoPC(15:0), methionyl-hydroxyproline, linoleamide, SM(d18:1/22:0), L-lysine, palmitic amide. While the significantly 11 metabolites were up-regulated and 8 metabolites were down-regulated. As shown in Fig. 4, clustering heatmap analysis of 19 metabolites revealed the differences of relative value among control, model and Ginsenoside Rg1 group, which brightness change of color indicate the concentration changes of biomarker. It was not hard to see that 14 biomaker of them were regulted by Ginsenoside Rg1 treatment. Relative signal intensities of serum metabolites in three groups identified by UPLC-Q/TOF-MS mirrors the intensity of the regulating activity of Ginsenoside Rg1 (Fig. 5).

### Metabolic pathways and network establishment

**Figure 6:**
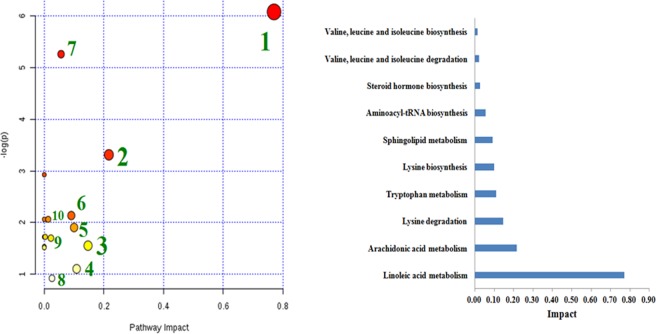
The metabolomic pathway and impact value analyzed by MetaboAnalyst related with Ginsenoside Rg1 treatment on AD. The impact value of pathway 1 to 10 are degressive.

**Figure 7:**
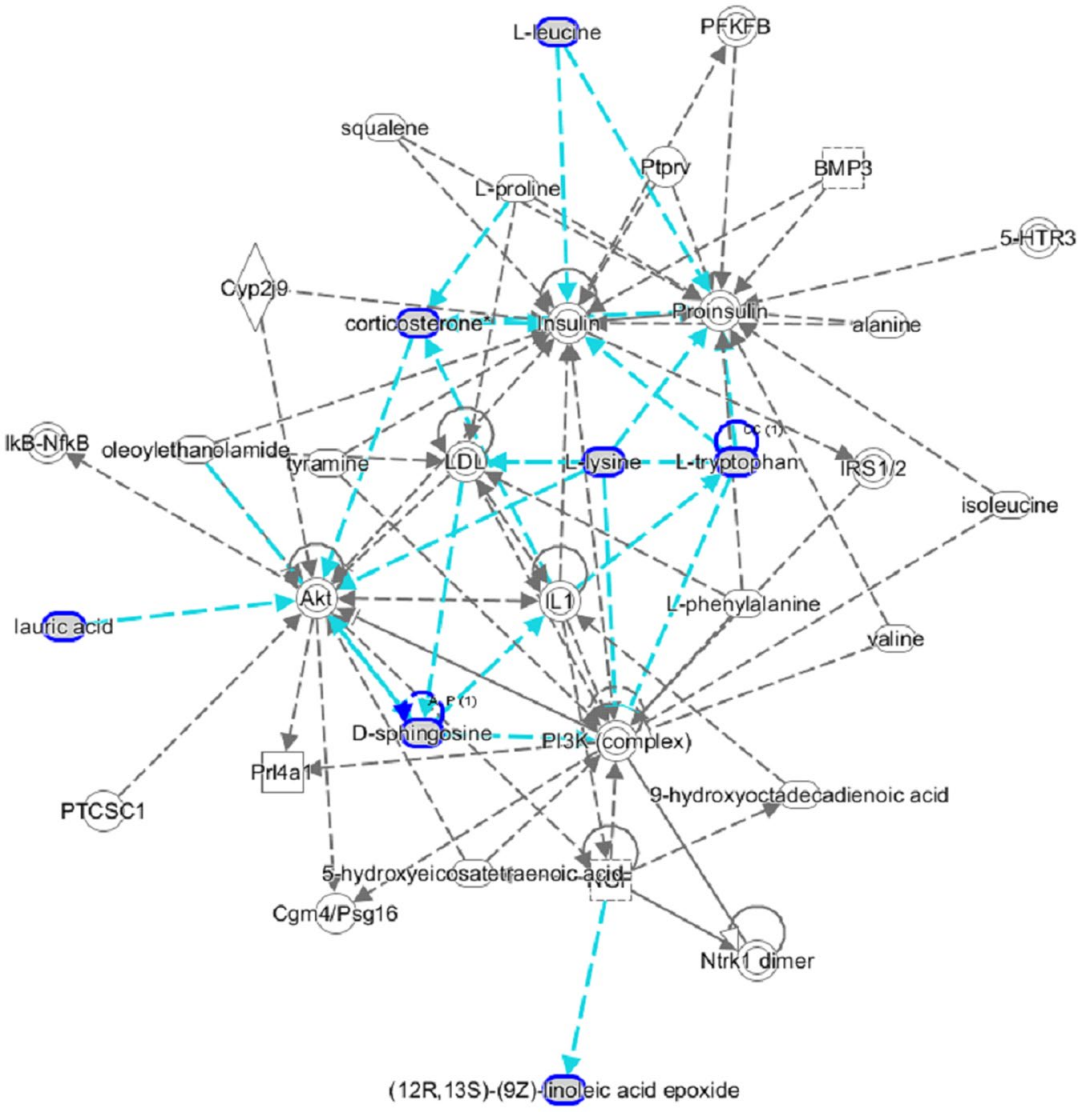
IPA prediction networks associated with Ginsenoside Rg1 protective activity on AD mice in this study.

Pathway topology analysis based on identified endogenous metabolites was built using MetPA as shown in Fig. 6. The possible pathways of selected metabolic biomarkers disposed by Ginsenoside Rg1 treatment close to normal mainly referrs to linoleic acid metabolism, arachidonic acid metabolism, lysine metabolism, tryptophan metabolism, sphingolipid metabolism, aminoacyl-tRNA biosynthesis, steroid hormone biosynthesis, valine, leucine and isoleucine metabolism. The linoleic acid, arachidonic acid, lysine degradation and tryptophan metabolism with p value of 0.77, 0.22, 0.14 and 0.10, respectively, were acutely regulted by Ginsenoside Rg1. Meanwhile, the information of these 14 metabolites were imported into IPA software to establish metabolic networks which is associated with Ginsenoside Rg1 treament (Fig. 7). The results showed that increased level of albumin, amino acid metabolism and molecular transport are main molecular mechanism of Ginsenoside Rg1 protecting against AD mice.

### Proteomics confirmation

**Figure 8:**
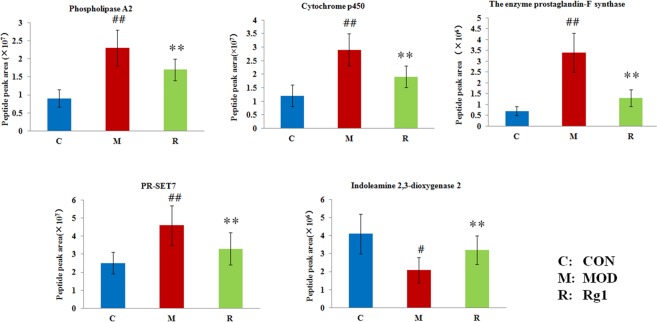
The changes of key enzymes analysis in metabolic pathways of AD mice using parallel reaction by target proteomics after Ginsenoside Rg1 treatment.

As shown in Fig. 8, the quantitative analysis of key enzymes in tangled metabolic pathways were employed by targeted proteomics for further verifying metabolomic result. Compared with control group, the protein expression of phospholipase A2 in linoleic acid metabolism, cytochrome p450 and the enzyme prostaglandin-F synthase in arachidonic acid metabolism and PR-SET7 in lysine degradation were markedly higher in model group (P < 0.01), meanwhile, indoleamine 2,3-dioxygenase 2 in tryptophan metabolism was also higher than in model group (P < 0.05). Compared with model group, all the specific enzyme were regulated by Ginsenoside Rg1, the changed trend was distinctly close to normal state (P < 0.01).

## Discussion

Animal models play an important role in the AD research process. It is also the main task of researchers to continuously explore better animal models. With the appearance of 3xTg-AD mice, the research on AD is more in-depth. It was reported that APP Swe and tau P301L were simultaneously performed to knock into PS1 M146V single cell embryos by microinjection for obtaining triple transgenic mice-3xTg-AD mice with APP Swe, tau P301L, PS1 M146V gene^28^. Compared with other AD mouse models, this model has many advantages, which it helps to clarify the relationship between Aβ, neurofibrillary tangles and synaptic dysfunction and is suitable for studying AD pathological changes in therapy. As a homozygous mouse, a well-defined offspring can be obtained without a large amount of analysis. Also, the heterozygote also undergoes neuropathological changes, and the penetrance of the gene phenotype is 100% with equal expressed between the male and female. Compared to the two transgenic mice, the 3xTg-AD mice model containing three transgenic genes has a single genetic background avoiding biological differences. As the animal model closest to the family-type AD, it has the main neuropathological features of AD-SP and NFT, and important pathological changes in AD such as neuronal death and synaptic loss in the brain^29^. The emergence of cognitive impairment and early pathogenesis of 3xTg-AD mice make the research process more economical and rapid. Currently, it is mainly used for pharmacodynamic evaluation and pharmacological studies of drugs, which have complete pathological findings in Aβ and tau proteins and are suitable for most anti-AD drugs. In addition, this model is also applicable to drug target studies such as mitochondria, synaptic damage, inflammation^30,31^.

Linoleic acid, 9(10)-EpOME and DHA were involved in linoleic acid metabolism in AD. Some studied were suggested that linoleic acid as a polyunsaturated essential fatty acids with neuroprotective activity could hold back the uptake of cholesterol and the cytotoxicity of Aβ42 in drosophila model of AD^32,33^. The increased accumulation of Aβ induces the production of free radicals, which resulting in the increase of lipid peroxidation in the brain. Linoleic acid also restrain the polymerisation of tau and Aβ. DHA possesses the property of scavenging free radicals for protecting against the peroxidation of protein and lipid in brains, reducing neuronal loss and cognitive deficits in ischemia–reperfusion brain injury animal models. The declining level of DHA gives rise to memory impairment and learning disability in aged organism^34^. 9(10)-EpOME is produced by linolenic acid peroxidation that the increased level of the oxidized low-density lipoprotein is closely associated with aging, rheumatoid arthritis, and atherosclerosis. In our research, the content of phospholipase A2 is increased to induce the up-regulation of linoleic acid and 9(10)-EpOME in model group, which is implicated the initiation of the inflammatory response in AD mice together with its lysophospholipid activity and the emergence of an immune response^35^. To linoleic acid metabolism, Ginsenoside Rg1 effectively enhance the DHA content and contribute to decrease the activity of linoleic acid and 9(10)-EpOME, which restore the impaired antioxidant function and senile plaques of brain in AD mice. Oxidative stress and inflammation are closely associated with AD morbidity. Arachidonic acid and 11b-PGF2a were involved in arachidonic acid metabolism in AD from our study result. Arachidonic acid could synthesize various bioactive substances such as LTA4, throm-boxane as well as prostaglandins and regulate leukocyte chemotaxis and inflammatory cytokine production which lead to inflammation reaction and organs dysfunction. During the entire inflammatory process, the increase level of reactive oxygen species (ROS) can not do without PGF2α that is an inflammatory mediator in cell. The high concentration and high consumption of oxygen in polyunsaturated fatty acids is extremely easy to cause oxidative damage to the brain^36,37^. Compared with model group, the abnormal concentration of arachidonic acid and 11b-PGF2a were called back, and the cytochrome p450 and the enzyme prostaglandin-F synthase are down-regulated Ginsenoside Rg1 treatment, which indicate that Ginsenoside Rg1 effectively reduce oxidative stress and inflammation reaction in AD mice. As a precursor of serotonin, tryptophan is an essential amino acid in protein synthesis, neurotransmitter conduction, and memory impairment^38^. Some reports were suggested that the increasing breakdown of tryptophan in peripheral blood of AD illness is closely related to chronic immune activation. Abnormal levels of lysine have been found in patients with Parkinson’s, hypothyroidism, kidney disease, asthma and depression^39–41^. The exact significance of these levels is unclear, yet lysine therapy can normalize the level and has been associated with improvement of some patients under these conditions. Compare with model group, the higher level of tryptophan and lysine were down-regulated by Ginsenoside Rg1 therapy, in addition, the level of PR-SET7 and indoleamine 2,3-dioxygenase 2 enzyme in lysine degradation and tryptophan metabolism were close to normal state by Ginsenoside Rg1 administration.

Metabolomics is an emerging life science discipline that analyzes abnormal metabolic pathways and related pathophysiological changes by probing into metabolite changes of body in an inverse mode^42–46^. After the Chinese medicine entering the human body, various biological pathways were regulated, which provides us the clues about the mechanism of drug action and the information of the corresponding parts^47–53^. In recent years, the prevention and treatment of AD based on the neurotoxicity of Aβ has progressed rapidly, and reports have gradually increased^54^. However, the current metabolomics research on the prevention and treatment of AD by Chinese medicine is still in its infancy. With the development of science and technology, combining metabolomics and TCM to study AD prevention and control strategies will be a hot research topic in the future. Metabolomics is performed to screen diagnostic markers for AD, improve the sensitivity and accuracy of diagnosis, and monitor the development of AD. Meanwhile, it attempts to discover novel TCM that effectively treat AD and explore its mechanism of action for promoting the early diagnosis of AD and interventional drug development.

## Conclusion

In our study, a UPLC-Q/TOF-MS-based serum metabolomics strategy has been employed to probe into the metabolic changes of triple transgenic AD mice in response to Ginsenoside Rg1 treatment for detailed research the protective activity of Ginsenoside Rg1 and pharmacodynamic mechanism. The results of non-targeted metabolomics showed that several metabolism pathways such as linoleic acid metabolism, arachidonic acid metabolism, lysine degradation, tryptophan metabolism and sphingolipid metabolism were involved in Ginsenoside Rg1 treatment. Significant changes of fourteen metabolites such as linoleic acid, 9(10)-EpOME, arachidonic acid, 11b-PGF2a, sphingosine and pyruvate were regulated by natural product administration and quantify in AD mice. These findings enhance our understanding of the protective action mechanism of Ginsenoside Rg1 and contribute to further optimizing of Ginsenoside Rg1 and its derivatives as anti-AD drugs.

## Supplementary information

SI
