# Supplementary material for: High-throughput metabolomics and ingenuity pathway approach reveals the pharmacological effect and targets of Ginsenoside Rg1 in Alzheimer’s disease mice
Source: Sci Rep. 2019 May 7;9:7040. doi: 10.1038/s41598-019-43537-4 (PMC6504884; doi:10.1038/s41598-019-43537-4)
Supplement: Supplementary file 1 — SI [file 41598_2019_43537_MOESM1_ESM.doc]

**High-throughput metabolomics and ingenuity pathway approach reveals the pharmacological effect and targets of Ginsenoside Rg1 in Alzheimer’s disease mice**

Ge Li^1^, Ning Zhang^3^, Fang Geng^4^, Guoliang Liu^3^, Bin Liu^3^, Xia Lei^3^, Guang Li^3^, Xi Chen^1,2^ ^*^.

1. Yunnan Branch, Institute of Medicinal Plant Development, Chinese Academy of Medical Sciences & Peking Union Medical College, Beijing, China;

2. Institute of Medicinal Plant Development, Chinese Academy of Medical Sciences & Peking Union Medical College, Beijing 100193, China

3.College of Jiamusi, Heilongjiang University of Chinese Medicine, Jiamusi, Heilongjiang 154007, Beijing, China

4.College of Chemistry & Chemical Engineering, Harbin Normal University, Harbin, Heilongjiang 150025, Beijing, China

Address correspondence to:

*Prof. Xi Chen

1. Yunnan Branch, Institute of Medicinal Plant Development, Chinese Academy of Medical Sciences & Peking Union Medical College, Beijing, China;

2. Institute of Medicinal Plant Development, Chinese Academy of Medical Sciences & Peking Union Medical College, Beijing, China.

Email: chenmetabolites@163.com

**Table S1** The information of serum markers change of AD mice before and after Ginsenoside Rg1 treatment

| No. | Ion form | Rt(min) | m/z | Proposed compound | Formula | Trend in model | Regulated |
| --- | --- | --- | --- | --- | --- | --- | --- |
| 1 | M-H | 3.83 | 279.23 | Linoleic acid | C18H32O2 | ↑ | ■ |
| 2 | M-H | 4.61 | 295.23 | 9(10)-EpOME | C18H32O3 | ↑ | ■ |
| 3 | M+H | 6.46 | 285.17 | Dihydroartemisinin (DHA) | C15H24O5 | ↓ | ■ |
| 4 | M-H | 3.98 | 303.23 | Arachidonic acid | C20H32O2 | ↓ | ■ |
| 5 | M+H | 2.71 | 355.25 | 11b-PGF2a | C20H34O5 | ↑ | ■ |
| 6 | M+H | 7.98 | 300.29 | Sphingosine | C18H37NO2 | ↑ | ■ |
| 7 | M-H | 1.98 | 115.04 | Pyruvate | C5H8O3 | ↑ | ■ |
| 8 | M-H | 0.81 | 130.09 | L-Leucine | C6H13NO2 | ↑ | ■ |
| 9 | M-H | 0.45 | 187.11 | Glycyl-Isoleucine | C8H16N2O3 | ↑ |  |
| 10 | M-H | 8.72 | 453.28 | Ursocholic acid | C24H40O5 | ↓ | ■ |
| 11 | M+H | 2.56 | 347.22 | Corticosterone | C21H30O4 | ↑ | ■ |
| 12 | M+H | 2.39 | 205.09 | L-Tryptophan | C11H12N2O2 | ↑ | ■ |
| 13 | M+H | 7.80 | 223.17 | Dodecanoic acid | C12H24O2 | ↓ | ■ |
| 14 | M+H | 10.06 | 482.32 | LysoPC(15:0) | C23H48NO7P | ↓ | ■ |
| 15 | M+H | 1.24 | 263.11 | Methionyl-Hydroxyproline | C10H18N2O4S | ↑ |  |
| 16 | M+H | 9.11 | 280.26 | Linoleamide | C18H33NO | ↓ |  |
| 17 | M-H | 2.78 | 785.65 | SM(d18:1/22:0) | C45H91N2O6P | ↓ |  |
| 18 | M+H | 4.07 | 147.11 | L-Lysine | C6H14N2O2 | ↑ | ■ |
| 19 | M+H | 8.52 | 256.26 | Palmitic amide | C16H33NO | ↓ |  |
